# Parasitic Infection and Host Immunity, 2nd Edition

**DOI:** 10.3390/microorganisms13061258

**Published:** 2025-05-29

**Authors:** Celio Geraldo Freire-de-Lima

**Affiliations:** Instituto de Biofísica Carlos Chagas Filho, Universidade Federal do Rio de Janeiro, Rio de Janeiro 21941-170, Brazil; celio@biof.ufrj.br

Parasitic infections arise when organisms such as protozoa, bacteria, fungi, or helminths invade a host to exploit its biological resources for survival and replication. The host immune system plays a pivotal role in detecting and neutralizing these invasions [1]. Innate immunity represents the first line of defense, deploying physical barriers, phagocytic cells, and pro-inflammatory mediators to limit the initial spread of parasites [2]. When these primary defenses are circumvented, the adaptive immune system is activated, initiating antigen-specific mechanisms such as antibody production and T cell-mediated responses [3]. Nevertheless, many parasites have evolved sophisticated immune evasion strategies, ranging from antigenic variation to active immunosuppression, enabling persistent infections and impeding immune clearance [4,5,6,7]. A deeper understanding of the intricate immunological interplay between host and parasite is fundamental for the development of targeted therapeutic approaches and effective vaccines.

This Special Issue presents a curated collection of scientific articles exploring key aspects of parasite–host interactions, with particular emphasis on the complex interplay between diverse parasitic organisms and host immune systems. Among these contributions is a discussion of a cornerstone concept in immunology: immunological tolerance, the immune system’s capacity to prevent responses against self-antigens. Immunological tolerance is crucial for maintaining immune homeostasis and preventing autoimmune diseases [8,9]. Rather than representing a mere absence of immune activity, tolerance is an active, tightly regulated process operating across multiple developmental stages and within distinct compartments of the immune system.

Two primary forms of immunological tolerance are recognized: central and peripheral. Central tolerance occurs in primary lymphoid organs (the thymus for T lymphocytes and bone marrow for B lymphocytes), where lymphocytes with high autoreactive potential are either eliminated or directed toward regulatory functions. This process eliminates most autoreactive lymphocytes during early development [10]. In contrast, peripheral tolerance functions outside primary lymphoid organs, utilizing mechanisms such as anergy, clonal deletion, and regulatory T-cell-mediated suppression to maintain tolerance to peripheral tissues and prevent autoimmunity [11].

During infection, many microorganisms exploit the immune system through molecular mimicry, a prominent immune evasion mechanism whereby pathogen-derived antigens closely resemble host molecules. While aiding immune evasion, molecular mimicry may inadvertently trigger autoimmune responses. The perspective article published by Martins and collaborators (Contribution 1) provides an insightful discussion on the role of molecular mimicry in parasitic infections, highlighting the challenge of maintaining immunological tolerance in its presence.

Chagas disease, caused by the protozoan *Trypanosoma cruzi*, is a neglected tropical disease affecting millions across Latin America, including women of reproductive age. Vertical transmission from mother to child occurs in approximately 4–8% of pregnancies among infected women [12]. However, the potential for *T. cruzi* transmission via breastfeeding remains under debate. Although *T. cruzi* DNA has been detected in the breast milk of infected mothers, confirmed cases of transmission through breastfeeding are exceedingly rare. Current clinical guidelines, including those issued by the World Health Organization (WHO), do not recommend discontinuing breastfeeding in *T. cruzi*-positive mothers, except in cases involving cracked or bleeding nipples, where the risk of transmission through blood contamination may increase. Further research is necessary to clarify the conditions under which breast milk-mediated transmission could occur and to safeguard maternal and infant health. A recent study by Cristóstomo-Vázquez et al. (Contribution 3) reported the detection of *T. cruzi* DNA in the breast milk of lactating women and the blood of their neonates, providing probable evidence of transmission through breastfeeding.

Another clinically significant protozoan parasite within the Trypanosomatidae family is *Leishmania*, a member of the Kinetoplastida order and the etiological agent of leishmaniasis. This disease is transmitted via the bite of phlebotomine sandflies and manifests in three major clinical forms: cutaneous, mucocutaneous, and visceral leishmaniasis (kala-azar), the latter being the most severe, and potentially fatal if untreated. It is estimated that over 1 billion people live in endemic areas, with 700,000 to 1 million new cases reported globally each year [13].

Distinct transcriptional profiles identified across the clinical spectrum of *Leishmania* (L.) *chagasi* infection reinforce the clinical–immunological framework that proposes five evolutionarily distinct stages of infection in endemic regions. The study by Da Matta et al. (Contribution 4) comprehensively characterized transcriptional alterations in the peripheral blood of infected individuals, revealing gene expression signatures that may enhance our understanding of the immunobiology of American visceral leishmaniasis, including asymptomatic and oligosymptomatic manifestations.

Another significant contribution focuses on *Leishmania braziliensis* infection. The manuscript by Leite-Silva and collaborators (Contribution 5) describes the upregulation of Toll-like receptor 4 (TLR4) in skin biopsies from patients with cutaneous leishmaniasis compared to uninfected controls. The authors suggest that this increased expression may be associated with interleukin-15 (IL-15) production. Interactions between infected dendritic cells (DCs) and natural killer (NK) cells are known to promote DC maturation, enhancing the expression of key migratory and co-stimulatory molecules (CCR7, CD40, CD80, CD83) and the secretion of pro-inflammatory cytokines such as interleukin-6 (IL-6). These processes culminate in interferon-gamma (IFN-γ) production by NK cells, amplifying effector responses and leukocyte activation. The data highlight the DC–NK cell axis as a key contributor to cutaneous leishmaniasis pathogenesis and propose CCR7 as a potential biomarker for disease progression monitoring.

The manuscript submitted by Benghu and collaborators (Contribution 6) presents a compelling study employing a coinfection model to elucidate the immunological interplay between tuberculosis (TB) and helminth infections. Their findings demonstrate that TB infection amplifies pro-inflammatory Th1 responses [14], whereas concomitant helminth infection markedly suppresses these responses [15]. Helminth infections, whether occurring independently or in conjunction with TB, are associated with enhanced Th2 activation and elevated regulatory cytokine production [16]. These observations underscore the capacity of helminths to drive a dominant Th2/Treg immune profile, which may compromise the Th1-mediated immunity essential for the effective control of Mycobacterium tuberculosis. This immunomodulatory effect carries significant implications for TB pathogenesis and may influence vaccine efficacy, particularly in helminth-endemic regions.

In parallel, Nunes and collaborators (Contribution 7) described that the pathogenesis of cutaneous leishmaniasis (CL) is marked by skin ulcers characterized by a localized inflammatory response and mediated by both innate and adaptive immune cells, including dendritic cells (DCs) and natural killer (NK) cells. Emerging evidence suggests that bidirectional interactions between DCs and NK cells play a pivotal role in determining the outcome of leishmaniasis. Despite substantial progress in understanding Leishmania biology [17], the precise mechanisms governing DC/NK cell-mediated control of Leishmania spp. pathogenesis, as well as the cellular and molecular mediators involved in these interactions, remain incompletely defined. In the aforementioned study, the authors investigated canonical pathways associated with CL resulting from Leishmania braziliensis infection. Through the analysis of two publicly available microarray datasets derived from skin biopsies of active CL lesions, five key pathways were identified based on differentially expressed genes. Among these, the “Crosstalk between DCs and NK cells” pathway emerged as particularly significant due to the large number of modulated genes. Critical molecules implicated in this pathway, including TLR4, TNFRSF1B, IL-15, IL-6, CD40, CCR7, TNF, and IFNγ, were identified and subsequently validated in newly collected CL biopsy samples. Notably, increased expression of CCR7 was found to correlate with lesion development [18], highlighting the “crosstalk between DCs and NK cells” as a promising pathway for further investigation in the pathogenesis of CL.

In another contribution, Scovino and collaborators (Contribution 7) provided important insights into SARS-CoV-2, the etiological agent of COVID-19 and a member of the *Betacoronavirus* genus. Focusing on the B.1 and P.1 variants, the study found that while these variants do not significantly exacerbate the cytokine storm associated with severe disease, the B.1 variant induces more substantial early cellular damage. A detailed structural and functional analysis of the spike protein revealed its central role in viral infectivity and immune evasion, with specific mutations enhancing ACE2 receptor binding affinity or reducing immune recognition. These findings highlight the critical importance of ongoing genomic surveillance of emerging SARS-CoV-2 variants and underscore the need for continued research into spike protein mutations to guide vaccine development and therapeutic strategies [19].

In conclusion, this Special Issue compiles a diverse array of studies that collectively advance our understanding of parasite–host interactions. Through experimental models and clinical investigations, these contributions illuminate the pathogen-specific nature of host immune responses, and the multifaceted challenges associated with infection control, diagnostics, and therapeutic interventions in parasitic and co-infectious diseases.

## Data Availability

Not applicable.
